# Assessing the Lethality of Suicide Attempts: Adding Chance of Rescue to Medical Severity

**DOI:** 10.1111/sltb.70058

**Published:** 2025-10-14

**Authors:** Tormod Stangeland, Ketil Hanssen‐Bauer, Linn‐Ingunn Lynum, Karen Margrethe Walaas Nedberge, Johan Siqveland

**Affiliations:** ^1^ Division of Mental Health and Addiction Services Akershus University Hospital Lørenskog Norway; ^2^ Clinic for Health Service Research and Psychiatry, Institute for Clinical Medicine, Medical Faculty University of Oslo Oslo Norway; ^3^ Department of Pediatric and Adolescent Mental Health Akershus University Hospital Lørenskog Norway; ^4^ National Centre for Suicide Research and Prevention, Institute of Clinical Medicine University of Oslo Oslo Norway

**Keywords:** adolescents, assessment, Fearlessness about Death, Interpersonal Theory of Suicide, lethality, Risk‐Rescue Rating Scale, suicide attempt

## Abstract

**Introduction:**

Emphasis on medical severity when assessing the lethality of suicide attempts may overlook important contextual factors. We examined if distinguishing between medical severity and chance of rescue improves evaluation and understanding of suicidal mental states.

**Methods:**

Seventy adolescent inpatients with a recent suicide attempt were interviewed with the Suicide Intent Scale, and clinicians rated the Risk‐Rescue Rating Scale, which provides separate ratings for medical severity (Risk) and chance of rescue (Rescue). They also completed the Interpersonal Needs Questionnaire and Fearlessness about Death scale.

**Results:**

The lethality components Risk and Rescue were uncorrelated (*r* = −0.02). However, Rescue was significantly negatively correlated with suicidal intent (*r* = −0.46), fearlessness about death (*r* = −0.29), and unmet interpersonal needs (*r* = −0.28), while Risk was only correlated with suicidal intent (*r* = 0.29). In a hierarchical regression model, Rescue was the strongest predictor of suicidal intent.

**Conclusion:**

Rescue factors, more than medical severity factors, were consistently related to our measures of suicidal mental state. Including the rescue component in lethality assessments may improve both the accuracy of clinical evaluations and our understanding of adolescents' mental state during suicidal crisis.

## 1 Introduction

Assessing the lethality of recent suicide attempts is essential to clinical care. Higher lethality predicts future high‐lethality suicide attempts (Rojas et al. 2019) and eventual death by suicide (Bergen et al. 2012; Runeson et al. 2010). Highly lethal suicide attempts warrant closer monitoring and more safety measures, such as inpatient treatment and increased follow‐up, to prevent further suicidal behavior. In cases where immediate security concerns are lower, there is more opportunity to explore the patient's state of mind and factors leading up to the suicide attempt.

### 1.1 Medical Severity as a Narrow Interpretation of the Lethality of Suicide Attempts

In the assessment of a suicide attempt, “lethality” often refers narrowly to indicators of medical harm or severity, such as physical injury, loss of consciousness, and the need for medical intervention (Beautrais 2003; Horesh et al. 2012). For example, the scale “Actual Lethality/Medical Damage” in the Columbia Suicide Severity Rating Scale (Posner et al. 2011) uses the term “lethality” as synonymous with medical damage.

However, a meta‐analysis of suicide attempts (Barker et al. 2022) found no association between the medical severity of suicide attempts and core clinical factors such as alcohol use, depression, cognitive functioning, aggression, or impulsivity. Only increased suicide intent and planning differentiated between high and low medical damage outcomes. In sum, medical severity appears to offer little insight into a suicidal patient's mental state.

### 1.2 A Broader View of Lethality as Danger to Life

We argue that focusing only on medical severity neglects other aspects that reduce or increase the danger of an attempt, which should be included in the lethality concept. Our argument aligns with Aaron Beck's broader definition of lethality in suicidology: “Lethality is the danger to life resulting from a suicide attempt” (Beck et al. 1975).

In our view, a complete lethality assessment should account for the circumstances surrounding the suicide attempt, which affect the probability of being rescued through the intervention of others. For example, the ingestion of an overdose of pills should be seen as far less lethal if the person alerts family members immediately after ingestion. Conversely, the focus on medical severity may underestimate the lethality of a suicide attempt if a method associated with lower risk (such as self‐poisoning) is combined with extensive precautions against rescue, such as leaving one's phone at home and going deep into unfamiliar woods. While these aspects are acknowledged in commonly used instruments for assessing suicidal behavior (Posner et al. 2011; Fox et al. 2020), they are rarely studied as a distinct aspect of lethality on equal terms with medical severity. In our clinical experience, they are also underutilized in assessment. We believe that knowing the circumstances surrounding a person's suicide attempt may give us insight into the person's state of mind and motivation for suicidal behavior, which cannot be derived from medical severity alone.

This distinction may also be useful for understanding how suicide attempts relate to central theoretical constructs in suicidology. Suicidal intent, defined as the intensity of the wish of a patient to terminate his life (Beck et al. 1974), is distinct from, yet related to, lethality. However, the relation is unclear. Patients who report higher suicidal intent after a suicide attempt have sometimes, but not always, made more lethal suicide attempts (Gjelsvik et al. 2017a; Persett et al. 2022), and they have a greater risk of eventual fatality by suicide (Harriss and Hawton 2005).

Similarly, in the Interpersonal Theory of Suicide (Joiner 2005; Van Orden et al. 2010), suicidal desire, when combined with acquired capability for suicide, is expected to lead to a higher risk of suicide attempt. A key aspect of acquired capability for suicide is developing fearlessness about death, a construct that has received much research attention. However, empirical findings are inconclusive regarding fearlessness about death as a mediator between suicidal desire and suicide attempts (Schmeckenbecher et al. 2024), even though it seems to have a small but significant predictive ability for the lethality of future suicide attempts (Ferm et al. 2020; Krantz et al. 2022; Shahnaz et al. 2020). According to the theory, pain tolerance and practical capability—such as access to and knowledge of lethal means also influence suicidal behavior, but these factors are not as well explored as fearlessness about death (Shahnaz et al. 2020).

In the research referred to above, lethality is typically measured by a single item and treated as a dichotomous variable of high or low lethality. A broader model of lethality and intent facilitates viewing suicide attempts as varying combinations of ambivalence, danger, and efforts to either prevent or invite rescue.

### 1.3 Examining Suicidal Adolescents From a Broader Perspective of Lethality

We propose that viewing suicide attempt lethality as a compound of two components (e.g., as measured by the Risk and Rescue components of the Risk‐Rescue Rating Scale (Weisman and Worden 1972)) may clarify its association with central constructs in suicidology and offer a better link for understanding suicidal states of mind. In our project, we were particularly interested in suicidal intent (as measured by the Suicide Intent Scale, SIS), because it, like lethality, describes a suicide attempt. We were also interested in unmet interpersonal needs that give rise to suicidal desire (as measured by the Interpersonal Needs Questionnaire, INQ), and fearlessness about death (as measured by Fearlessness about Death, FAD).

Our aim was to compare adolescents who made suicide attempts of varying medical severity and under different circumstances of potential rescue, and to examine how these conditions relate to suicidal intent, fearlessness about death, and unmet interpersonal needs.

- Research question 1: Do the Risk and Rescue components show distinct associations with SIS, INQ, and FAD, compared to the overall RRRS?
- Research question 2: Does including both the Risk and Rescue components account for additional variance in SIS in a hierarchical linear regression model?

We expect that models using either an overall measure of lethality (RRRS) or solely a narrow measure of medical severity (Risk) will result in a low association with suicidal intent, fearlessness about death, and unmet interpersonal needs. However, by including both the components of suicide lethality (Risk and Rescue), we expect to find a stronger association between these measures.

## 2 Methods

### 2.1 Participants

We recruited adolescents admitted to inpatient treatment following a suicide attempt. From September 2022 to March 2025, we invited patients referred to two departments at a major Norwegian hospital after a suicide attempt to participate in the study:

1. adolescents admitted to the acute department of the Child and Adolescent Mental Health Services (CAMHS), and
2. adolescents assessed by the CAMHS liaison team at the hospital's somatic emergency department.

Taken together, these two departments assess nearly all adolescents who come into contact with CAMHS after a suicide attempt in the hospital's catchment area. An exception includes a small number of patients discharged from the somatic emergency department following brief admissions at nighttime or weekends outside the service hours of the liaison team, and who were not subsequently referred to the psychiatric acute ward. We expect these patients without follow‐up to have made less severe suicide attempts or to have a limited need for acute mental health services.

Exclusion criteria included adolescents with ongoing conflict regarding treatment measures or hospitalization, overt hostility or lack of communication, insufficient proficiency in both Norwegian and English, or high anxiety or confusion that would make research participation unethical or infeasible.

### 2.2 Measurements

The Risk‐Rescue Rating Scale (RRRS) (Weisman and Worden 1972) is a clinician‐rated instrument based on observable rating criteria, selected to minimize the need for subjective interpretation. Five items constitute the Risk component, which assesses the suicide method used, the resulting medical damage, and level of treatment after a suicide attempt. Five items constitute the Rescue component, which assesses the location of the attempt, actions taken to avoid or elicit attention from rescuers, and the degree of familiarity of the person who initiated rescue. The RRRS and its two components were not developed to measure single latent traits, but rather as clinical indexes developed to capture distinct, complementary indicators of lethality.

Based on all available information from medical health records, witness reports, and the patient, a clinician rates each item on a three‐point scale. The Risk and Rescue components are combined in a short assessment of the event—for instance, “a suicide attempt of low medical risk, with a high chance of rescue”—a format suited for clinical communication. The components' ratings may also be aggregated into a numerical total rating for the overall assessment of lethality, for monitoring changes in a single patient's suicidal behavior, or for comparing patient groups. Because of the instrument's rating structure, a high Rescue rating is beneficial, indicating lower lethality and a greater chance of survival. For the other measures in the study, including Risk, high scores indicate higher lethality or greater distress, resulting in negative correlations with the Rescue component.

A Norwegian version has been developed and tested, showing promising interrater reliability (ICC 0.93) and concurrent validity with the Suicide Intent Scale (Stangeland et al. 2025).

The Suicide Intent Scale (SIS) (Beck et al. 1974) is a semi‐structured interview designed to assess a patient's preparation and intent to die by suicide, as well as the patient's expectation, ambivalence, and possible wish to die through the suicide attempt. Medical severity is not part of the SIS rating. Instead, the interview aims to capture the patient's perspective of the suicide attempt and rate intent based on their descriptions. The authors comment that patients' reports are subject to distortion, and that rather than disqualifying reports with inconsistent validity, the examiner should describe the process and make contextual notes to support the assessment. A clinician rates 15 items on a three‐point scale, and the SIS is the sum of all the item ratings with a range from 0 to 30. We have tested the interrater reliability of SIS and found an ICC of 0.94 (Stangeland et al. 2025).

Norwegian version of the SIS has been used in several studies (Gjelsvik et al. 2017a; Grøholt et al. 2000; Hjelmeland et al. 1998; Persett et al. 2022).

The Interpersonal Needs Questionnaire (INQ) (Van Orden et al. 2012) is a self‐report questionnaire based on the Interpersonal Theory of Suicide (IPTS) (Joiner 2005) which posits that suicidality is deeply connected to unmet interpersonal needs. The Interpersonal Needs Questionnaire measures the subject's experience of thwarted belongingness and perceived burdensomeness. According to the theory, the combined presence of both these constructs is the proximal cause of a desire for suicide. The instrument is rated through self‐report on items ranging from 1 to 7. We chose the 12‐item version, since it has performed well in use with adolescents (Quan et al. 2021) and is available in a Norwegian version.

Fearlessness about Death (FAD): According to the IPTS, suicidal behavior requires the acquired ability to overcome instinctive fear and avoidance of danger. This may be assessed through the Acquired Capability for Suicide Scales, used most often in its revised version, the questionnaire Fearlessness about Death (Ribeiro et al. 2014). It consists of seven self‐report items ranging from 0 to 4.

We translated the questionnaire into Norwegian using the back‐translation procedure. Five experienced clinicians—highly familiar with suicide prevention and the target population and with high proficiency in both English and Norwegian independently translated the material. They compared their versions and discussed discrepancies until they reached consensus on the most appropriate translation. Two professionals then back‐translated the consensus version into English. The consensus version and notes on the translation process were reviewed by an external bilingual professional writer with a PhD in the Social Sciences, and his comments were incorporated into the final version.

Electronic patient record data were collected to register demographic information, diagnostic assessments, and reports from acute mental health care episodes and assessments of previous suicidal events. We also registered Children's Global Assessment Scale (CGAS), a brief measure of psychosocial functioning (range 1–100) (Shaffer et al. 1983).

### 2.3 Procedure

Health personnel at the two participating hospital departments routinely administer RRRS and SIS as part of their assessment of suicide attempts. After intake assessment, eligible patients were invited to participate in the study. The participants completed the INQ and FAD at the first appropriate opportunity following inclusion.

We manually extracted data from electronic patient health records to register the participants' history of suicide attempts and diagnostic assessments.

### 2.4 Analysis

We used SPSS version 30 to conduct all statistical analyses, including descriptives of central statistics for all the study measures. Data from all participants was used for correlation analysis for INQ, FAD, SIS, RRRS and its components Risk and Rescue in answer to Research question 1. In answer to Research question 2, we conducted a hierarchical linear regression with SIS as the dependent variable, adding first INQ and FAD, followed by Risk and finally Rescue. Age, gender, and the interaction term for INQ/FAD were tested but did not significantly improve model fit and were excluded from the final model. To check for the effect of delay between suicide attempt and testing, we reran all tests without participants with more than one month delay and found no difference in descriptives or analysis outcomes. All tests were considered significant for *p*‐values < 0.05.

For participants with a history of multiple suicide attempts, we examined the most recent attempt.

## 3 Results

A total of 85 adolescents referred after suicide attempts agreed to participate in the study. Of these, 10 adolescents were excluded because they could not be assessed with RRRS or SIS shortly after the suicide attempt, and 5 because of incomplete or invalid RRRS or SIS data. Two INQ and FAD protocols had to be discarded because the responses were clearly invalid, leading to 68 complete protocols and two partial cases describing suicide attempts only.

Routine data from the clinic indicates that the participants had similar demographic and diagnostic characteristics and similar treatment duration to the overall patient population at the clinic. Detailed comparisons between participants and non‐participants could not be made, as we collected no information about the non‐participants.

Table 1 presents demographic and diagnostic information about the participants at the time of assessment.

**TABLE 1:** Characteristics of the sample: Demographics and diagnostic assessment at time of inclusion.

	*n*
Total participants	70
Age (SD) in years	15.6 (1.5)
Age range in years	12–18
Female (%)	57 (81%)
Mean days passed since most recent suicide attempt	5.26
Median days passed since most recent suicide attempt	1
CGAS mean (SD)	51.0 (7.0)

Primary ICD‐10 diagnostic assessment at time of inclusion	*n*
Psychoactive substance use disorder	4
Depressive disorder	11
Anxiety disorder	17
Hyperkinetic disorder/ADHD	6
Other mental and behavioral disorder^a^	5
Symptom diagnosis^b^	27
Total	70

Abbreviations: CGAS, Children's Global Assessment Scale; SD, standard deviation.

^a^ F50 anorexia nervosa, F60.9 unspecified personality disorder, F84.5 Asperger syndrome, and F94.1 reactive attachment disorder of childhood.

^b^ R41 Other symptoms and signs involving cognitive functions and awareness and R45 symptoms and signs involving emotional state.

The participants' age ranged from 12 to 18 years, with a median age of 16. The mean Children's Global Assessment Scale (CGAS) rating at intake was 51.0, indicating moderate impairment in psychosocial functioning. Many of the participants had only brief hospital admissions focused on acute crisis stabilization, leading to 27 cases of symptom diagnosis (R‐diagnosis in ICD‐10 chapter XVIII) only.

Sixty‐eight of the 70 participants had made a recent suicide attempt (within the last 30 days), two within the last two months. Mean time from suicide attempt to assessment was 5.3 days and median 1 day. The most common method was self‐poisoning, with 56 of the 70 registered attempts. The remaining attempts involved strangulation, hanging, cutting, and leaping from heights or into traffic, with three or four cases of each.

Table 2 presents descriptive statistics for the study measures. The self‐report scales INQ and FAD showed good internal consistency and variability. The clinician‐rated SIS showed strong internal consistency. Internal consistency was not calculated for RRRS and its components, as they reflect distinct clinical indicators.

**TABLE 2:** Descriptives for INQ, FAD, SIS, RRRS and its components, risk and rescue.

Measure	*N*	Mean (SD)	Range	Cronbach's alpha
INQ	68	4.40 (1.14)	1.0–6.9	0.87
FAD	68	2.89 (0.78)	0.6–4.0	0.74
SIS	70	12.41 (6.50)	0–25	0.85
RRRS	70	31.77 (12.01)	17–75	N/A^a^
Risk	70	7.13 (1.54)	5–13	N/A^a^
Rescue	70	12.67 (2.08)	7–15	N/A^a^

Abbreviations: FAD, Fearlessness about Death; INQ, Interpersonal Needs Questionnaire; N/A, not applicable; RRRS, Risk‐Rescue Rating Scale; SD, standard deviation; SIS, Suicide Intent Scale.

^a^ Index of distinct factors, Cronbach's alpha is not psychometrically meaningful.

Table 3 presents the individual items in the RRRS and its two components, with brief item descriptions. There were no extreme or outlying item means, but it should be noted that Rescue item 1 (“Location remoteness”) had a 91.4% frequency of its max value, indicating that nearly all the suicide attempts in the sample occurred in familiar or accessible locations.

**TABLE 3:** Distribution of values for risk and rescue items (%).

	1	2	3
Risk items			
1: Method used	84.3	4.3	11.4
2: Reduced consciousness	75.7	20.0	4.3
3: Resulting damage	47.1	40.0	12.9
4: Treatment duration	82.9	15.7	1.4
5: Treatment level	32.9	62.9	4.3
Rescue items			
1: Location remoteness	1.4	7.2	91.4
2: Relation with rescuer	12.9	11.4	75.7
3: Attention from others	20	31.4	48.6
4: Eliciting rescue	17.1	20.0	62.9
5: Time before rescue	15.7	28.6	55.7

Answering Research question 1, Table 4 presents Pearson correlations between the study measures, including the Risk and Rescue components of the RRRS. The RRRS total was significantly correlated with SIS (*r* = 0.43) but showed no meaningful correlation with either INQ or FAD.

**TABLE 4:** Correlations between the two RRRS components and study measures.

	Risk	Rescue	RRRS	SIS	INQ	FAD
Risk	—					
Rescue	−0.02	—				
RRRS	0.66**	−0.67**	—			
SIS	0.29*	−0.46**	0.43**	—		
INQ	0.03	−0.28*	0.14	0.32**	—	
FAD	−0.19	−0.29*	0.11	0.12	0.30*	—

*Note: N*s range from 68 to 70 due to missing data for INQ and FAD.

Abbreviations: FAD, Fearlessness about Death; INQ, Interpersonal Needs Questionnaire; RRRS, Risk‐Rescue Rating Scale; SIS, Suicide Intent Scale.

* *p* < 0.05.

** *p* < 0.01.

The Risk component, representing medical severity, demonstrated a similar but slightly weaker correlation with SIS (*r* = 0.29), and it remained uncorrelated with INQ and FAD.

The Rescue component, representing situational factors, showed a distinct profile, significantly (negatively) correlated not only with SIS (*r* = −0.46), but also with INQ (*r* = −0.28) and FAD (*r* = −0.29). There was no correlation between Risk and Rescue (*r* = −0.02), underscoring their conceptual and empirical independence.

**FIGURE 1:**
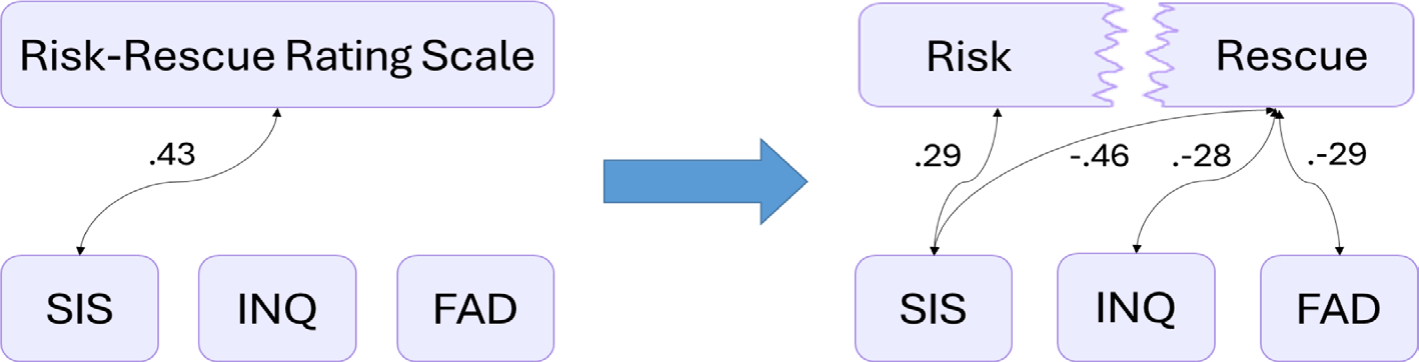
Correlations for the Risk‐Rescue Rating Scale (RRRS) and its two components. *Left panel*: Significant correlations (*p* < 0.05) between the overall RRRS and the other study measures. *Right panel*: Significant correlations (*p* < 0.05) between the RRRS components—Risk and Rescue—and the same measures. FAD, Fearlessness about Death; INQ, Interpersonal Needs Questionnaire; SIS, Suicide Intent Scale.

Figure 1 illustrates these findings graphically, showing only the significant correlations displayed in Table 4, between the RRRS and the study measures, compared to the Risk and Rescue correlations with the same measures. This figure highlights the nuances absent from the overall lethality measure.

To answer Research question 2 and examine how the two components of the RRRS contribute to understanding suicidal intent, we conducted a hierarchical linear regression with SIS rating as the dependent variable. The model is presented in Table 5.

**TABLE 5:** Hierarchical linear regression analysis of predictors of SIS.

Hierarchical models	*R* ^2^	Adjusted *R* ^2^	*R* ^2^ change	SE of the estimate	*F*	Sig.	*F* change	Sig. *F* change
1 Predictors: INQ, FAD	0.104	0.077	0.104	6.257	3.787	0.028	3.787	0.028
2 Predictors: INQ, FAD, risk	0.19	0.152	0.086	5.996	5.003	0.004	6.764	0.012
3 Predictors: INQ, FAD, risk, rescue	0.323	0.280	0.133	5.524	7.524	< 0.001	12.41	0.001

Final model coefficients	Unstandardized coefficients		Standardized coefficients	*t*	Sig.
	*B*	SE	Beta		
3 Predictors: FAD, INQ, risk, rescue					
(Constant)	14.69	7.481		1.964	0.054
INQ	1.177	0.64	0.205	1.84	0.07
FAD	−0.051	0.952	−0.006	−0.053	0.958
Risk	1.146	0.442	0.275	2.593	0.012
Rescue	−1.218	0.346	−0.391	−3.523	0.001

*Note:* No standardized residuals exceeded ±2.1, and the highest Cook's distance was 0.079, indicating no influential outliers. Collinearity diagnostics indicated no issues; all VIF values were below 1.25 and all tolerance values exceeded 0.80.

Abbreviations: FAD, Fearlessness about Death; INQ, Interpersonal Needs Questionnaire; SIS, Suicide Intent Scale.

Our primary interest in this analysis was not in maximizing explained variance, but in evaluating the added value of specifying the Risk and Rescue components. In the first step, a baseline model including INQ and FAD explained only a small proportion of the variance in SIS. In the second step, Risk was added, resulting in a statistically significant increase in explained variance (Δ*R* ^2^ = 0.086, Δ*F*(1, 63) = 6.76, *p* = 0.012). In the final step, Rescue was entered into the model. This addition yielded a clear improvement in model fit (Δ*R* ^2^ = 0.133, Δ*F*(1, 63) = 12.41, *p* < 0.001), and Rescue emerged as the strongest individual predictor of suicidal intent (std *β* = −0.39, *p* < 0.001).

When age and gender were entered into an exploratory model, they had no significant contribution (Δ*R* ^2^ = 0.046, Δ*F*(2, 63) = 1.70, *p* = 0.191) and were excluded from the final model for clarity.

In another exploratory model, we added the Risk*Rescue interaction term. This showed a trend‐level association with SIS (*B* = −0.413, SE = 0.210, *β* = −0.217, *p* = 0.053), indicating that the association between Risk and SIS was strongest when Rescue values were low. In this model, the main effect was reduced for Risk (std *β* = 0.219) and slightly increased for Rescue (std *β* = −0.435).

## 4 Discussion

In answer to our research questions, adolescents who made suicide attempts under conditions with a lower chance of rescue and greater medical severity tended to have higher levels of suicidal intent, but only rescue factors were related to fearlessness about death and unmet interpersonal needs.

### 4.1 Risk and Rescue as Separate Factors for Understanding Suicidal Mental States

We found that the Risk component was associated with intent. However, the study measures of interpersonal needs and fearlessness about death showed no significant relation to the medical severity of the attempts, as measured by Risk. The Rescue component had stronger associations with all the study measures than the Risk component did. Additionally, Rescue showed a stronger association with suicide intent than Risk in our regression model. An exploratory, non‐significant interaction effect between Risk and Rescue further increased the relative influence of Rescue. In sum, medical severity may be less informative about the mental state of the adolescents who attempt suicide. Adolescents who take deliberate steps to avoid discovery and interruption tend to report greater suicidal intent, more unmet interpersonal needs, and greater fearlessness about death.

This is in line with Sapyta et al. (2012), who found no relationship between medical lethality and suicide intent in a group of adolescents, in contrast to a stronger relationship for young adults. Yang et al. (2022) contribute an interesting nuance to this topic, finding that the relationship was moderated by the accuracy of the person's expectation of death. This indicates that knowledge of suicidal methods and understanding of the means of suicide may be important for understanding the suicidal process. This underscores the importance of a person's mental state when assessing suicide attempts.

It is somewhat unexpected that we found no significant association between Risk and Rescue. To our knowledge, only one other study has reported on this relationship in adolescents (Rengasamy et al. 2020), and their findings indicated a strong significant association. However, their sample consisted of adolescents who had made medically serious suicide attempts, which may have restricted the range of Risk scores and contributed to the observed association. We suspect that the lack of research on this topic stems from the tradition of treating the circumstances of suicide attempts as a minor variable that is not typically measured separately or available for study.

According to the Interpersonal Theory of Suicide, a person's mental state drives the suicidal process, and FAD and INQ play central roles in the model. However, Ferm et al. (2020) and Krantz et al. (2021) found no significant difference in FAD between the groups with and without suicide attempts. Similarly, in Schmeckenbecher et al.'s meta‐analysis (2024), FAD did not differentiate between suicide attempts and non‐suicidal self‐injury. If fearlessness about death is not higher among persons who make suicide attempts, decisions about suicide attempts may be due to idiosyncratic and situational factors. However, the Interpersonal Theory of Suicide defines Acquired Capability for Suicide to also encompass pain tolerance, knowledge, and access to lethal methods. These moderating factors may influence the suicidal process, but remain less operationalized for empirical study (Shahnaz et al. 2020).

We argue that a model of the path from mental state to suicidal behavior should avoid restricting itself to a dichotomous classification of adolescents with and without suicide attempts. Knowing not only *whether* a person attempted suicide, but *how* it was done and *why* it was interrupted may offer better insight into the interplay between suicidal desire, intent, and capability.

In view of the Interpersonal Theory's concept of Acquired Capability, adolescents as a group may lack the knowledge or means to carry out medically severe suicide attempts, even if they express fearlessness and high suicidal intent. This interpretation is supported by the fact the Risk and Rescue levels of suicide attempts in our sample have relatively low lethality, as were the ratings in another RRRS study of inpatient adolescents (Grøholt et al. 2000), compared to Risk ratings in adult patients (Berardelli et al. 2021; Kim et al. 2021). Similarly, both our sample and Grøholt et al.'s adolescent sample (2000) showed higher Rescue values than the adult samples. Self‐poisoning was the dominant method, and nearly all cases in our sample took place in familiar locations with a high chance of being discovered and rescued.

### 4.2 Implications for the Assessment of Suicide Attempts

Because lethality plays a central role in decisions about treatment and safety measures following a suicide attempt, the choice of sources of information included in its assessment has significant implications for clinical practice.

First, the current emphasis on medical severity contains a risk of overlooking relevant information from the circumstances surrounding the attempt. This may lead clinicians to either overestimate or underestimate suicide lethality: A medically severe attempt interrupted by the patient's self‐rescue should be considered as less lethal than a medically less dangerous attempt in which the patient took deliberate steps to avoid discovery. Misinterpreting an attempt as highly lethal could lead to interventions that are unnecessarily focused on immediate safety, instead of the underlying psychological distress leading to the suicidal crisis.

Second, examining the circumstances of the attempt may provide a better understanding of patients' state of mind than focusing solely on medical risk. The components Risk and Rescue were uncorrelated in our study, suggesting that they reflect different aspects of suicide attempts. Risk provided little insight into the adolescents' mental state, while Rescue showed a meaningful correspondence. These contextual aspects of suicide attempts are topics clinicians could tap into to initiate meaningful therapeutic conversations with suicidal patients about their suicidal desire.

Third, we found that medical severity was lower and the chance of rescue was higher in our sample than in comparable samples of adults. Safety measures are often called for when assessing adolescents' suicidal behavior, typically by monitoring or restricting their activities to prevent suicidal behavior. While some adolescents experience this as helpful, it is also a common cause of conflict and distraction from the efforts to help with the underlying causes of the behavior. Our findings indicate that immediate safety measures often are less pressing for adolescents than establishing a therapeutic alliance and understanding their situation.

### 4.3 Strengths and Limitations

The study design combines the mental health and somatic departments of a major hospital catchment area, ensuring that nearly all the adolescents assessed at the hospital level for acute mental health crisis were eligible for inclusion. This enhances the potential generalizability of our findings.

The acute setting enabled testing shortly after a suicide attempt, with a median interval of only one day from the attempt to assessment. As self‐report from such events tends to shift over time (Gjelsvik et al. 2017b), we see the immediate testing as a clear advantage when attempting to probe the state of mind of suicide attempters.

However, many eligible adolescents declined participation. Because we could not record health information about non‐participants, we have no record of their suicide attempts or whether they differ from the participants in fearlessness about death or unmet interpersonal needs. A larger sample would have allowed more robust statistical analyses, particularly in the regression model (Table 5), where the interaction term could be examined with greater confidence.

A very high percentage of participants in our sample were female. While this reflects the gender distribution typically seen in acute mental health services, it raises the question of how these findings might generalize to populations with a more balanced gender composition. Berardelli et al. (2022) found differences in Risk and Rescue ratings between men and women with suicidal attempts. Given that constructs such as suicidal intent, fearlessness about death, and interpersonal needs may vary between genders, future studies should investigate whether the associations observed here hold in more diverse samples.

Several health personnel contributed assessment data to the study, and we have no direct measure of their interrater reliability. A recent study involving the same groups of health personnel rating theoretical cases indicated excellent reliability for lethality and intent (Stangeland et al. 2025), but we cannot know how this would apply to real cases.

Adolescents tend to engage in less serious suicide attempts than those of adults. While we believe our findings are representative of adolescents, the relatively low lethality in our sample yielded lower variability in the data than we would expect if high‐lethality samples were included. This may have limited the sensitivity of our analysis. A larger study involving more patients and data from distinct groups would give a better base from which to generalize our findings.

## 5 Conclusion

In our study of adolescent suicide attempts, the chance of rescue, more than medical severity, was consistently related to our measures of suicidal mental state. Including the rescue component in lethality assessments may improve both the accuracy of clinical evaluations and our understanding of adolescents' mental state during suicidal crisis.

## Author Contributions

**Tormod Stangeland:** conceptualization, methodology, software, data curation, formal analysis, project administration, validation, visualization, investigation, writing – original draft, writing – review and editing, resources, funding acquisition. **Ketil Hanssen‐Bauer:** methodology, data curation, formal analysis, validation, supervision, writing – original draft, writing – review and editing. **Linn‐Ingunn Lynum:** data curation, validation, methodology, investigation, writing – original draft, writing – review and editing, visualization. **Karen Margrethe Walaas Nedberge:** data curation, methodology, validation, investigation, writing – original draft, writing – review and editing, visualization. **Johan Siqveland:** conceptualization, methodology, data curation, supervision, formal analysis, validation, investigation, funding acquisition, visualization, project administration, resources, writing – original draft, writing – review and editing.

## Ethics Statement

The study is part of a broader project approved by the Norwegian Regional Ethics Committee (REK approval number 322341) and by the hospital's Data Protection Officer.

## Consent

Informed consent was obtained from all participants and from the parents or guardians of participants below 16 years of age. No adverse patient‐related events were registered during data collection.

## Conflicts of Interest

The authors declare no conflicts of interest.

## Data Availability

The study design and analysis were not preregistered. All data, analysis codes, and research materials are available upon request from the corresponding author. The data are not publicly available due to privacy or ethical restrictions.
